# Factorial Structure and Preliminary Validation of the Schema Mode Inventory for Eating Disorders (SMI-ED)

**DOI:** 10.3389/fpsyg.2018.00600

**Published:** 2018-04-24

**Authors:** Susan G. Simpson, Giada Pietrabissa, Alessandro Rossi, Tahnee Seychell, Gian Mauro Manzoni, Calum Munro, Julian B. Nesci, Gianluca Castelnuovo

**Affiliations:** ^1^School of Psychology, Social Work and Social Policy, University of South Australia, Adelaide, SA, Australia; ^2^Regional Eating Disorders Unit, St. John's Hospital, NHS Lothian, Livingston, United Kingdom; ^3^Psychology Research Laboratory, Ospedale San Giuseppe, IRCCS Istituto Auxologico Italiano, Verbania, Italy; ^4^Department of Psychology, Catholic University of Milan, Milan, Italy; ^5^Psychology Department, University of Adelaide, Adelaide, SA, Australia; ^6^Faculty of Psychology, eCampus University, Como, Italy; ^7^Department of Psychiatry, Royal Edinburgh Hospital, University of Edinburgh, Edinburgh, United Kingdom; ^8^Spectrum: Personality Disorder Service, Victoria, Melbourne, VIC, Australia

**Keywords:** factorial structure, psychometric properties, schema therapy, modes, assessment, eating disorders, eating behaviors

## Abstract

**Objective:** The aim of this study was to examine the psychometric properties and factorial structure of the Schema Mode Inventory for Eating Disorders (SMI-ED) in a disordered eating population.

**Method:** 573 participants with disordered eating patterns as measured by the Eating Disorder Examination Questionnaire (EDE-Q) completed the 190-item adapted version of the Schema Mode Inventory (SMI). The new SMI-ED was developed by clinicians/researchers specializing in the treatment of eating disorders, through combining items from the original SMI with a set of additional questions specifically representative of the eating disorder population. Psychometric testing included Confirmatory Factor Analysis (CFA) and internal consistency (Cronbach's α). Multivariate Analyses of Covariance (MANCOVA) was also run to test statistical differences between the EDE-Q subscales on the SMI-ED modes, while controlling for possible confounding variables.

**Results:** Factorial analysis confirmed an acceptable 16-related-factors solution for the SMI-ED, thus providing preliminary evidence for the adequate validity of the new measure based on internal structure. Concurrent validity was also established through moderate to high correlations on the modes most relevant to eating disorders with EDE-Q subscales. This study represents the first step in creating a psychometrically sound instrument for measuring schema modes in eating disorders, and provides greater insight into the relevant schema modes within this population.

**Conclusion:** This research represents an important preliminary step toward understanding and labeling the schema mode model for this clinical group. Findings from the psychometric evaluation of SMI-ED suggest that this is a useful tool which may further assist in the measurement and conceptualization of schema modes in this population.

## Introduction

Schema Therapy (ST) was developed to address long-standing psychological disorders and entrenched personality traits (Young et al., 2003), and a growing number of studies have demonstrated its effectiveness (Masley et al., 2012; Jacob and Arntz, 2013; de Klerk et al., 2016). ST is based on the notion that Early Maladaptive Schemas (EMS) develop as a result of the interaction between temperament and unmet core emotional needs during childhood. EMS consist of patterns of memories, cognitions, emotions and physical reactions that drive coping mechanisms, and become increasingly maladaptive over time. Whereas EMS refer to stable “traits,” schema modes represent the moment-to-moment “states” and coping responses that we all experience. Schema modes are traditionally grouped into four main categories: (1) Innate Child Modes, that represent the emotions experienced in the context of unmet needs; (2) Internalized/Introject [Parent] Modes, which represent the internalized messages from childhood, including parents, teachers, other caregivers, and culture; (3) Coping Modes, which are the survival mechanisms developed during childhood and adolescence to cope with unmet emotional needs, and (4) Adaptive (Healthy) Modes. In ST, the emphasis is on developing healthy coping mechanisms that facilitate the process of developing adaptive ways of meeting one's interpersonal and emotional needs, whilst weakening maladaptive modes and associated EMS (Young et al., 2003).

The (124-item) Schema Mode Inventory (SMI) (Young et al., 2007) was developed to measure schema modes through self-report. A shortened (118 item) version of the original SMI (Young et al., 2007) was validated within a sample of healthy controls, axis I and axis II patients (Lobbestael et al., 2010) and found to have acceptable internal consistency and test-rest reliability. A 14-factor model emerged, consisting of: five child modes, five dysfunctional coping modes, two dysfunctional parent modes and the adaptive Healthy Adult mode. The SMI was primarily developed to measure schema modes in the Borderline and Antisocial PDs, however, it has recently successfully been adapted (SMI-2) to more appropriately measure schema modes as they manifest within Cluster C and paranoid, histrionic and narcissistic PDs (Bamelis et al., 2011). Previous studies have highlighted the need for exploratory research that examines schema modes within specific clinical groups in order to begin to delineate the profiles and new sub-modes that may be identified in these populations (Lobbestael et al., 2010).

A significant proportion of those with Eating Disorders (EDs) do not respond or do not complete treatment (Waller et al., 2009) and go on to develop chronic symptoms (Keller et al., 2006; Castelnuovo et al., 2011). Rigid personality traits are common in this population, with up to 69% of the ED population meeting criteria for a co-morbid personality disorder (PD) (Blinder et al., 2006), a factor that has been shown to adversely affect treatment outcomes (Grilo et al., 2007; Sallet et al., 2010; Farstad et al., 2016). In spite of improved outcomes with Enhanced CBT (CBT-E) (Fairburn et al., 2008; Castelnuovo et al., 2017; Pietrabissa et al., 2017), it remains less effective at addressing rigid co-morbid personality characteristics, such as perfectionism and avoidant traits (Byrne et al., 2011). The Schema Therapy Model for EDs has evolved over the past 10 years (Waller, 2003; Waller et al., 2007; Simpson, 2012), prompted by the need for therapeutic approaches that address deeper level cognitions and entrenched behaviors that do not respond to first-line treatments (Waller, 1997; Waller and Kennerley, 2003; Sorgente et al., 2017).

Preliminary research suggests that Schema Therapy may be highly suited to the needs of those with EDs, particularly those with high levels of complexity or chronicity (Simpson et al., 2010; Simpson and Slowey, 2011; McIntosh et al., 2016). This approach goes beyond maintenance factors to address the core schema-level beliefs that underpin ED psychopathology. Preliminary studies in this area suggest that those with EDs may experience significantly higher levels of maladaptive modes than community samples (Voderholzer et al., 2014; Talbot et al., 2015). Preliminary conceptualizations of the ED population suggest that all three coping styles (avoidance, surrender and overcompensation) play a significant role in driving and perpetuating ED behaviors (Waller and Kennerley, 2003; Simpson, 2012). Recent evidence supports the notion that maladaptive coping modes mediate the relationship between perceived negative parenting and eating disorder symptoms (Sheffield et al., 2009; Brown et al., 2016). In particular, the “Eating Disorder Overcontroller (Coping) Mode (EDO)” has been proposed as highly relevant to the ED population in recent theoretical conceptualizations (Simpson, 2012, 2016), and supported by findings from a recent study (Brown et al., 2016). This mode is theoretically linked to the Unrelenting Standards schema, and functions as a form of overcompensation, whereby perfectionism, achievement, and competitiveness, often focused on the body and eating patterns, are utilized to provide distance from underlying feelings of vulnerability and distress by generating a sense of competence, omnipotence and control.

The EDO has some parallels with the “Perfectionistic Overcontroller” mode that has been identified as highly relevant within Obsessive-Compulsive Personality Disorder (Bamelis et al., 2011), which has high comorbidity with EDs (Halmi et al., 2005). However, in eating disorders, it is proposed that the EDO has a more substantial focus on controlling the body, attaining a state of purity, and an emotional “high” associated with overcoming basic human needs (Simpson, 2016). Although the EDO is tapping into a similar construct to that of other clinical perfectionism measures, it is focused on specific characteristics and behaviors of the ED population, and focused on a broader concept than that measured by these other scales. In addition, the Helpless Surrenderer has been proposed as a mode which has particular relevance to those with EDs. In this mode the patient avoids expressing vulnerability or emotional needs directly, but instead seeks help passively, such as through withdrawal, complaint, or seeking “quick-fix” solutions. This mode may be mistaken for the Vulnerable Child mode due to what appears to be overt distress. It is hypothesized that this mode may be closely linked with Dependence, Emotional Deprivation, and Subjugation schemas (Simpson, 2016).

The purpose of this study was to develop a psychometrically sound adapted version of the Schema Mode Inventory in order to facilitate more precise measurement of mode states within a population with self-reported disordered eating behaviors. The result, a new Schema Mode Inventory for Eating Disorders (SMI-ED) would contribute to the ST conceptualization of schema modes in this sample. More specifically, this study aimed to conduct a Confirmatory Factorial Analysis (CFA) in order to examine the structure and psychometric properties of the SMI-ED, and to determine the internal reliability of its subscales. The relationship between eating disorder symptoms (restraint, binge-eating, and purging) and schema modes was also explored. In addition, we investigated whether specific EDE-Q subscales discriminated schema modes.

## Materials and methods

### Participants

Between February 2014 and April 2015 participants were recruited through advertisements placed on Facebook and websites of various local and international not-for-profit eating disorder organizations and support groups. Websites that advertised the survey to members included the Butterfly Association in Australia, and BEAT in the United Kingdom. Specifically, a small paragraph invited those with symptoms of disordered eating to take part in the study. In addition, flyers were placed around University campuses and in clinical waiting rooms of local eating disorder services in South Australia. The first page of the survey provided participants with details of the study and ethics approval to enable them to give informed consent.

The study was open to individuals aged between 18 and 70, who were English-speaking with disordered eating. Selection criteria were chosen in order to capture a wide sample of participants with any disordered eating, including those with subthreshold eating disorders. Our screening criteria were based on previous research (Mond et al., 2004, 2006) based on a sample of young Australian women. We used their 75th percentile as a cut-point for the global Eating Disorder Examination Questionnaire (EDE-Q 6.0) (Fairburn and Beglin, 2008) score, indicating “probable eating disorder case.” However, we extended their criteria to additionally include participants who reached the 75th percentile on any of the 4 EDE-Q subscales. Specifically, to be included in the final data set, participants were required to meet at least one of the 2 inclusion criteria: (Young et al., 2003) a global EDE-Q score of 2.3 or higher, in conjunction with repeated bingeing episodes and/or use of exercise or other compensatory behavior over the past 4 weeks (Mond et al., 2004, 2006); and/or (de Klerk et al., 2016) a mean score at or above the 75 percentile on at least one of the EDE-Q subscales—Restraint: 2.2; Eating Concern: 1; Weight Concern: 2.8; Shape Concern: 3.5.

A total of 672 people participated in the survey. Of these, 45 participants did not complete the survey and were excluded from the final analyses. Of the remaining 573 participants, 519 met all of the recommended inclusion criteria of the study, and 54 scored at or above the 75% percentile rank on at least one EDE-Q subscale. The final sample comprised 573 participants [33 males (5.8%) and 540 females (94.2%)] aged from 18 to 61 years (mean = 27.08, SD = 8.84, *95%CI*_*mean*_ = from 26.35 to 27.81). Participants' self-reported BMI ranged from 12.07 to 85.44 (mean = 23.43; SD = 8.60), with 25.7% of the sample under 18.5. 353 subjects were single, 100 in a de facto relationship, and 88 were married. Of the total sample, 36.1% of the total sample had been educated to at least year 12 of high school, while 28.8% were university graduates and 13.6% had a postgraduate degree. The majority of the sample was Australian (62.8%), and 93.4% of the total sample spoke English as their first language.

Of the 573 participants, 201 reported that they had been diagnosed by a mental health professional with Anorexia Nervosa (AN), 74 with Bulimia Nervosa (BN), 21 with Binge Eating Disorder (BED), and 97 with Eating Disorders Not Otherwise Specified (EDNOS). The remaining 180 participants had not received a formal diagnosis and/or had not consulted a mental health professional. Descriptive statistics are presented in Table 1. Diagnoses could not be verified by the researchers due to the online nature of this study, therefore Table 1 refers to EDE-Q subscales.

**Table 1:** Descriptive statistics for the EDE-Q subscales.

	**Overall sample** (***n*** = **573)**		**Restraint** (***n*** = **130)**		**Eating concern** (***n*** = **298)**		**Shape concern** (***n*** = **77)**		**Weight concern** (***n*** = **68)**		Statistics^*^	***p***
Weight—In *kg* (*mean*; *SD*)	65.14	25.17	50.15	13.98	65.92	22.47	86.45	31.56	66.26	26.46	*K* = 15.86	0.001
Height—in *m* (*mean*; *SD*)	1.66	0.11	1.67	0.07	1.67	0.13	1.67	0.08	1.66	0.08	*K* = 0.21	0.977
BMI (*mean*; *SD*)	23.54	8.59	18.18	4.52	23.86	7.62	30.93	10.75	23.01	9.13	*K* = 153.72	0.001
Age (*mean*; *SD*)	27.08	8.84	27.62	9.27	26.13	7.77	31.36	11.23	25.32	7.82	*K* = 173.68	0.001
Gender (*n*; %)											*V* = 0.153	
Male	33	5.8%	4	3.1%	13	4.4%	66	85.7%	63	92.6%		
Female	540	94.2%	126	96.9%	285	95.6%	11	14.3%	5	7.4%		
Relationships status (*n*; %)											*V* = 0.130	
Single /Never married	353	61.6%	82	63.1%	195	65.4%	39	50.6%	37	54.4%		
Married	88	15.4%	17	13.1%	39	13.1%	20	26.0%	12	17.6%		
In a de-facto relationship	100	17.5%	20	15.4%	54	18.1%	9	11.7%	17	25.0%		
Separated	11	1.9%	5	3.8%	3	1.0%	2	2.6%	1	1.5%		
Divorced	17	3.0%	5	3.8%	6	2.0%	6	7.8%	0	0%		
Widowed	4	0.7%	1	0.8%	1	0.3%	1	1.3%	1	1.5%		
Education status (*n*; %)											*V* = 0.105	
Year 10 of high school	16	2.8%	2	1.5%	13	4.4%	0	0%	1	1.5%		
Year 11 of high school	32	5.6%	11	8.5%	16	5.4%	4	5.2%	1	1.5%		
Year 12 of high school	207	36.1%	48	36.9%	111	37.2%	20	26.0%	28	41.2%		
TAFE/Trade certificate	75	13.1%	17	13.1%	38	12.8%	12	15.6%	8	11.8%		
Bachelor Degree	165	28.8%	38	29.2%	81	27.2%	24	31.2%	22	32.4%		
Postgraduate degree/PhD	78	13.6%	14	10.8%	39	13.1%	17	22.1%	8	11.8%		
Employment status (*n*; %)											*V* = 0.140	
Stay at home parent	14	2.4%	5	3.8%	5	1.7%	3	3.9%	1	1.5%		
Student	207	36.1%	41	31.5%	110	36.9%	27	35.1%	29	42.6%		
Part-time	123	21.5%	24	18.5%	60	20.1%	20	26.0%	19	27.9%		
Full-time	140	24.4%	29	22.3%	75	25.2%	21	27.3%	15	22.1%		
Unemployed	28	4.9%	4	3.1%	22	7.4%	1	1.3%	1	1.5%		
Sickness/disability pension	61	10.6%	27	20.8%	26	8.7%	5	6.5%	3	4.4%		
Mother language (*n*; %)											*V* = 0.075	
English	535	93.4%	124	95.4%	277	93.0%	96	89.6%	65	95.6%		
Non-English	38	6.6%	6	4.6%	21	7.0%	8	10.4%	3	4.4%		
Nationality (*n*; %)											*V* = 0.124	
Australian	360	62.8%	78	60.0%	185	62.1%	49	62.3%	49	72.1%		
British	79	13.8%	25	19.2%	42	14.1%	9	11.7%	3	4.4%		
American	64	11.2%	12	9.2%	35	11.7%	7	9.1%	10	14.7%		
New Zealander	8	1.4%	3	2.3%	3	1.0%	2	2.6%	0	0%		
Canadian	8	1.4%	2	1.5%	5	1.7%	0	0%	1	1.5%		
Hispanic	3	0.5%	0	0%	1	0.3%	2	2.6%	0	0%		
Chinese	5	0.9%	0	0%	2	0.7%	2	2.6%	1	1.5%		
Others	45	7.9%	10	7.7%	25	8.4%	7	9.1%	4	5.9%		
Location completing survey (*n*; %)										*V* = 0.085	
Australia	410	71.6%	88	67.7%	209	70.1%	60	77.9%	53	77.9%		
Other Country	163	28.4%	42	32.3%	89	29.9%	17	22.1%	15	22.1%		
Consulted MH professional - Past (*n*; %)											*V* = 0.189	
Yes	462	80.6%	117	90.0%	245	82.2%	53	68.8%	47	69.1%		
No	111	19.4%	13	10.0%	53	17.8%	24	31.2%	21	30.9%		
Consulted MH professional - Present (*n*; %)											*V* = 0.220	
Yes	227	48.3%	83	63.8%	147	43.9%	23	29.9%	24	35.3%		
No	296	51.7%	47	36.2%	151	50.7%	54	70.1%	44	64.7%		

* *Due to the strong different sample size in each group and some non-normal distributions of variables, Cramér's V and Kruskal-Wallis's test were performed to test potential associations across socio-demographics statistics*.

### Sample size calculation

Sample size was based on Comrey and Lee's (1992) recommendation that 500 or more observations can be considered “very good” for conducting factor analyses (MacCallum et al., 1999).

### Measures

#### Demographic survey

Prior to commencing the questionnaire, general demographic information was collected (weight in kg; height in meters; BMI; age; gender; relationships, education and employment status; mother language; nationality; location completing survey; current or past consultation with a mental health professional). Those who were not receiving support were invited to provide contact details so that they could be contacted if it was indicated that they would benefit from treatment.

#### The eating disorder examination questionnaire (EDE-Q)

The EDE-Q 6.0 (Fairburn and Beglin, 2008) is a 28-item self-report measure of the range and severity of ED features. The questions concern the frequency in which the patient engages in behaviors indicative of an eating disorder over a 28-day period. The test is scored on a 7-point scale from 0 to 6, rated using four subscales (Restraint; Eating concern; Shape concern, and Weight concern) and a global score. The EDE-Q has generally received support as an adequately reliable and valid measure of eating-related pathology and specific disordered eating behaviors (Peterson et al., 2007) and the scale has moderate to strong positive correlations with the gold standard Eating Disorder Examination structured interview (Berg et al., 2011). Similarly, in the present sample, the dimensions of the EDE-Q have demonstrated acceptable internal consistency (R-α = 0.828; EC-α = 0.811; SC-α = 0.894; WC-α = 0.831; General/Total-α = 0.942).

#### The schema mode inventory for eating disorders (SMI-ED)

The item-pool for the new SMI-ED was developed through combining selected items from the original SMI (Young et al., 2007), with a set of additional items (Table 2). The SMI-ED was constructed based on items derived from 3 main sources: (1) Items from the original SMI that were considered most relevant to those with EDs (n = 117); (2) A new set of items (n = 73) that were generated by clinicians/researchers specialized in the treatment of eating disorders, two psychologists (authors SS and JN) and one psychiatrist (author CM). All had experience in the field ranging from 6 to 20 years. Items were based on typical self-statements made by patients across all diagnostic groups and the Schema Therapy theoretical conceptualization of eating disorders (Simpson, 2016), and (3) Items put forward by patients with EDs who volunteered to contribute. Four patients (two with a diagnosis of AN, one with BED and one with BN) suggested items that were relevant to their most commonly experienced emotional and coping states. The patients also contributed to the way in which items were clustered together under specific mode categories. This was particularly the case for the “Helpless Surrenderer” mode, whereby the items were clustered together based on their experience of the different elements of this mode.

**Table 2:** Factor loadings (λ) and the explained variance (R^2^) of the SMI-ED.

**Factor**	**Item from the SMI-ED**	**λ**	**R^2^**
**VC**—**VULNERABLE CHILD**			
1	I feel fundamentally inadequate, flawed, or defective. (¤)	0.765	0.586
2	My behavior is out of control. (^*^)	0.618	0.382
3	I feel full/bloated/uncomfortable. (^*^)	0.571	0.327
4	Even if there are people around me, I feel lonely. (¤)	0.810	0.657
5	I am too greedy. (^*^)	0.500	0.250
6	I feel desperate. (¤)	0.806	0.650
7	In the end, my eating behaviors (i.e.: restriction, bingeing, purging, exercising) are the only ways of gaining some comfort. (^*^)	0.722	0.521
8	It is only when I am medically unwell that I feel it is legitimate for me to get the help I need. (^*^)	0.588	0.345
9	I feel ashamed of who I am. (^*^)	0.778	0.606
10	My body feels huge. (^*^)	0.646	0.418
11	I feel lonely. (¤)	0.844	0.712
12	If I lose control of my eating I feel unsafe. (^*^)	0.664	0.440
13	I feel left out or excluded. (¤)	0.737	0.543
14	My eating behavior and/or weight loss is the only way I get others to notice what I need. (^*^)	0.554	0.307
15	I feel lost. (¤)	0.835	0.697
16	I feel humiliated. (¤)	0.775	0.600
17	I feel that nobody loves me. (¤)	0.779	0.607
18	I often feel alone in the world. (¤)	0.844	0.713
19	Once I become distressed, my emotions feel out of control. (^*^)	0.754	0.569
20	I feel weak and helpless. (¤)	0.825	0.681
**AC**—**ANGRY CHILD**			
21	If someone is not with me, he or she is against me. (¤)	0.644	0.415
22	I feel enraged towards other people. (¤)	0.749	0.560
23	I have a lot of anger inside me that I can only soothe through my eating behaviors (e.g. restriction, bingeing, purging, exercising). (^*^)	0.755	0.570
24	I am angry that people do not understand me. (^*^)	0.836	0.699
25	I feel like telling people off for the way they have treated me. (¤)	0.771	0.595
26	I feel angry and resentful when others don't notice what I need. (^*^)	0.798	0.637
27	I'm angry that people are trying to take away my freedom or independence. (¤)	0.689	0.475
28	I have a lot of anger built up inside of me that I need let out. (¤)	0.859	0.738
29	I've been cheated or treated unfairly. (¤)	0.733	0.537
30	I feel like lashing out or hurting someone for what he/she did to me. (¤)	0.777	0.603
31	My eating behaviors (i.e.: restricting, bingeing, purging, exercising) are my way of getting back at others when I'm angry. (^*^)	0.635	0.403
32	I feel angry at others for making me feel hurt. (^*^)	0.813	0.661
33	I'm angry with someone for leaving me alone or abandoning me. (¤)	0.719	0.517
34	It makes me angry when someone tells me how I should feel or behave. (¤)	0.684	0.468
35	If I don't fight, I will be hurt, abused or ignored. (¤)	0.714	0.510
**EC**—**ENRAGED CHILD**			
36	I can become so angry that I feel capable of killing someone. (¤)	0.688	0.473
37	I destroy things when I'm angry. (¤)	0.823	0.677
38	I have rage outbursts. (¤)	0.884	0.782
39	I physically attack people when I'm angry at them. (¤)	0.633	0.400
40	My anger gets out of control. (¤)	0.876	0.767
41	When I'm angry, I often lose control and threaten other people. (¤)	0.797	0.635
42	I have been so angry that I emotionally hurt others (e.g. by shouting at him/her). (¤)	0.803	0.645
43	If I get angry, I can get so out of control that I injure other people. (¤)	0.597	0.356
**IC**—**IMPULSIVE CHILD**			
44	I say what I feel, or do things impulsively, without thinking of the consequences. (¤)	0.838	0.702
45	I break rules and regret it later. (¤)	0.787	0.619
46	It feels impossible for me to control my impulses. (¤)	0.821	0.674
47	I have trouble controlling my impulses. (¤)	0.805	0.649
48	I blindly follow my emotions. (¤)	0.808	0.653
49	I act first and think later. (¤)	0.862	0.742
50	I don't think about what I say, and it gets me into trouble or hurts other people. (¤)	0.776	0.601
51	If I feel the urge to do something, I just do it. (¤)	0.855	0.731
52	I act impulsively or express emotions that get me into trouble. (¤)	0.861	0.742
**UC**—**UNDISCIPLINED CHILD**			
53	I can't be bothered to finish tasks. (^*^)	0.781	0.610
54	I get fed up with rules and end up doing whatever I feel like. (^*^)	0.727	0.528
55	I don't discipline myself to complete routine or boring tasks. (¤)	0.814	0.662
56	I can't bring myself to do things that I find unpleasant, even if I know it is for my own good. (¤)	0.799	0.639
57	It's not worth the effort to plan how you'll handle situations. (^*^)	0.762	0.581
58	I'm lazy. (¤)	0.616	0.380
59	I get bored easily and lose interest in things. (¤)	0.730	0.533
60	If I can't reach a goal, I become easily frustrated and give up. (¤)	0.748	0.559
**HC**—**HAPPY CHILD**			
61	I feel that I fit in with other people. (¤)	0.667	0.445
62	I feel spontaneous and playful. (¤)	0.542	0.294
63	I trust most other people. (¤)	0.700	0.489
64	I feel safe. (¤)	0.791	0.625
65	I feel loved and accepted. (¤)	0.843	0.710
66	I feel optimistic. (¤)	0.797	0.635
67	I feel at peace on my own. (^*^)	0.579	0.336
68	I feel content and at ease. (¤)	0.836	0.698
69	I feel that I have plenty of stability and security in my life. (¤)	0.804	0.647
70	I feel connected to other people. (¤)	0.866	0.751
71	I feel listened to, understood, and validated. (¤)	0.849	0.721
**PM**—**PUNITIVE MODE**			
72	I can't forgive myself. (¤)	0.674	0.454
73	I am not allowed to bother others with my problems. (^*^)	0.721	0.520
74	I don't deserve to be alive. (^*^)	0.843	0.710
75	I'm pathetic. (^*^)	0.839	0.704
76	I'm angry at myself. (¤)	0.807	0.651
77	I deserve to be punished. (¤)	0.896	0.803
78	I'm a bad person if I get angry at other people. (¤)	0.779	0.606
79	I don't deserve anything that gives me pleasure (i.e.: eating, play, nurturance). (^*^)	0.897	0.805
80	I punish myself if I make a mistake. (¤)	0.833	0.694
81	It's my fault when something bad happens. (¤)	0.839	0.705
82	I'm a bad person. (¤)	0.904	0.817
83	If people could see the real me they would be disgusted. (^*^)	0.837	0.700
84	It feels right to deprive myself of normal things that people need (i.e.: food, closeness, love). (^*^)	0.881	0.777
85	I don't allow myself to do pleasurable things that other people do because I'm bad. (¤)	0.905	0.818
86	I have impulses to punish myself by hurting myself (e.g. cutting myself). (¤)	0.729	0.532
87	Others see the real me as undesirable. (^*^)	0.769	0.592
88	I don't deserve sympathy when something bad happens to me. (¤)	0.870	0.756
89	I deny myself pleasure because I don't deserve it. (¤)	0.898	0.807
90	If I stopped being hard on myself I'd be a terrible person. (^*^)	0.785	0.617
**DM**—**DEMANDING MODE**			
91	I have to be happy, smiling, outgoing and caring to be liked. (^*^)	0.596	0.356
92	I demand high standards of my body to avoid being judged. (^*^)	0.727	0.528
93	I find it difficult to accept anything less than perfection from myself. (^*^)	0.815	0.665
94	I must do things the right way to avoid criticism. (^*^)	0.824	0.678
95	I have to take care of the people around me. (¤)	0.614	0.377
96	I sacrifice pleasure, health, or happiness to meet my own standards. (¤)	0.833	0.693
97	I should be able to cope with everything myself and not need help. (^*^)	0.738	0.545
98	My life revolves around getting things done and doing them right. (¤)	0.808	0.653
99	I know that there is a ‘right’ and a ‘wrong’ way to do things; I try hard to do things the right way, or else I start criticizing myself. (¤)	0.825	0.680
100	At the end of the day, I will be judged by my body shape. (^*^)	0.603	0.363
101	I'm under constant pressure to achieve and get things done. (¤)	0.760	0.578
102	I'm pushing myself to be more responsible than most other people. (¤)	0.694	0.482
103	I don't let myself relax or have fun until I've finished everything I'm supposed to do. (¤)	0.707	0.500
104	I'm hard on myself. (¤)	0.665	0.443
105	I have to do things correctly and to perfection. (^*^)	0.844	0.712
**HA**—**HEALTHY ADULT**			
106	I know when to express my emotions and when not to. (¤)	0.363	0.132
107	I can find pleasure in simple things life offers (a sunset, a beautiful tree etc.). (^*^)	0.595	0.354
108	I can understand and form positive intimate relationships. (^*^)	0.682	0.466
109	I seek advice if I cannot solve problems on my own. (^*^)	0.615	0.379
110	I feel that I am basically a good person. (¤)	0.758	0.575
111	I value my body as an important part of me. (^*^)	0.574	0.330
112	I can stand up for myself when I feel unfairly criticized, abused or taken advantage of. (¤)	0.557	0.310
113	When necessary, I complete boring and routine tasks in order to accomplish things that I value. (¤)	0.458	0.209
114	I take care of my body and health. (^*^)	0.645	0.416
115	I assert what I need without going overboard. (¤)	0.671	0.451
116	I have a good sense of who I am and what I need to make myself happy. (¤)	0.781	0.610
117	When there are problems I try hard to solve them myself. (¤)	0.350	0.122
118	I can solve problems rationally without letting my emotions overwhelm me. (¤)	0.590	0.348
119	I'm capable of taking care of myself. (¤)	0.517	0.267
120	I can accept and enjoy praise and compliments. (^*^)	0.724	0.525
121	I feel able to learn, grow, and change. (^*^)	0.763	0.583
122	I accept myself body as it is. (^*^)	0.596	0.356
**CS**—**COMPLIANT SURRENDER**			
123	I let other people get their own way instead of expressing my own needs. (¤)	0.792	0.627
124	I act in a passive way, even when I don't like the way things are. (¤)	0.740	0.547
125	I allow other people to criticize me or put me down. (¤)	0.772	0.596
126	I need to compromise more than others. (¤)	0.769	0.591
127	I change myself depending on the people I'm with, so they'll like me or approve of me. (¤)	0.661	0.437
128	I prefer not to bother others with my feelings. (¤)	0.736	0.542
129	In relationships, I let the other person have the upper hand. (¤)	0.772	0.596
130	I can put up with anything from people who are important to me. (¤)	0.694	0.481
131	I try very hard to please other people in order to avoid conflict, confrontation or rejection. (¤)	0.774	0.600
132	In relationships, I have to give more to compensate for my lack of worth. (^*^)	0.790	0.623
**DET.PR**—**DETACHED PROTECTOR**			
133	I feel distant from other people. (¤)	0.783	0.612
134	I feel indifferent about most things. (¤)	0.754	0.569
135	It's more comfortable to keep my distance from others. (^*^)	0.760	0.577
136	Appearing sick/unwell to others helps me to avoid being responsible for things in my life. that I feel anxious about. (^*^)	0.530	0.280
137	I feel flat. (¤)	0.701	0.491
138	I don't feel connected to other people. (¤)	0.845	0.714
139	I feel cold and heartless toward other people. (¤)	0.671	0.450
140	I feel nothing. (¤)	0.831	0.690
141	If people try to come too close I keep them at a distance. (^*^)	0.784	0.615
142	I feel detached (no contact with myself, my emotions or other people). (¤)	0.846	0.716
143	I don't care about anything; nothing matters to me. (¤)	0.772	0.595
**DET.S-S**—**DETACHED SELF-SOOTHER**			
144	My eating behaviors (i.e.: restriction, bingeing, purging, exercising) help me to detach from difficult emotions. (^*^)	0.731	0.534
145	I keep busy in order to distract myself from unpleasant thoughts or feelings. (^*^)	0.688	0.473
146	I like doing something exciting or soothing to avoid my feelings (e.g., working, gambling, eating, exercise, shopping, sexual activities, watching TV). (¤)	0.533	0.285
147	I work or play sports intensively so that I don't have to think about upsetting things. (¤)	0.549	0.302
148	I want to distract myself from upsetting thoughts and feelings. (¤)	0.756	0.571
149	Being thin (or overweight) makes me invisible to people who might hurt me. (^*^)	0.620	0.384
**SA**—**SELF AGGRANDISER**			
150	I do things to make myself the center of attention. (¤)	0.485	0.235
151	I won't settle for second best. (^*^)	0.617	0.381
152	It's important for me to be Number One in some area of my life (e.g., the most popular, most successful, most wealthy, most powerful, thinnest). (^*^)	0.689	0.474
153	I get irritated when people don't do what I ask them to do. (¤)	0.684	0.469
154	I have to be the best in whatever I do. (¤)	0.655	0.428
155	I'm quite critical of other people. (¤)	0.697	0.486
156	I do what I want to do, regardless of other people's needs and feelings. (¤)	0.644	0.415
157	I feel I shouldn't have to follow the same rules that other people do. (¤)	0.661	0.437
158	Thinness is a way in which I can be better than others. (^*^)	0.640	0.410
159	I'm demanding of other people. (¤)	0.688	0.473
160	I feel special and better than most other people. (¤)	0.590	0.348
**BA**—**BULLY AND ATTACK**			
161	Equality doesn't exist, so it's better to be superior to others. (¤)	0.775	0.601
162	By dominating other people, nothing can happen to you. (¤)	0.818	0.669
163	I belittle others. (¤)	0.712	0.507
164	I feel invincible. (^*^)	0.470	0.221
165	If you don't dominate other people, they will dominate you. (¤)	0.827	0.684
166	I demand respect by not letting other people push me around. (¤)	0.657	0.432
167	I mock or bully other people. (¤)	0.592	0.350
168	If you let other people mock or bully you, you're a loser. (¤)	0.560	0.314
169	Attacking is the best defense. (¤)	0.693	0.480
170	If I'm not constantly on guard people will take advantage of me (^*^)	0.482	0.232
171	I always look for ways to outsmart others, to ensure that they cannot take advantage of me or hurt me in any way. (^*^)	0.646	0.417
**HS—HELPLESS SURRENDERER**			
172	I want people to understand me without me having to say anything. (^*^)	0.754	0.568
173	I need people to listen to me and make me feel better. (^*^)	0.743	0.552
174	It's too hard to make changes to my behavior. (^*^)	0.653	0.426
175	I feel angry and desperate when people can't see I need help. (^*^)	0.813	0.661
176	I am very possessive and hold on to the people that are important to me. (^*^)	0.471	0.222
**EDO**—**EATING DISORDER OVERCONTROLLER**			
177	Feeling in control of my eating ‘trumps’ any problems or disappointments going on in my life. (^*^)	0.815	0.665
178	Controlling my eating and/or exercise makes me feel powerful. (^*^)	0.726	0.527
179	I get satisfaction from adhering to the rules I set for myself. (^*^)	0.719	0.518
180	Controlling my eating/weight disconnects me from life's problems. (^*^)	0.780	0.609
181	I find it hard to let go and relax. (^*^)	0.867	0.752
182	If I'm in control of my eating, I can stay on top of my emotions. (^*^)	0.784	0.614
183	Controlling my eating gives me a physical and mental ‘high’. (^*^)	0.841	0.707
184	I take pride in controlling my weight and shape. (^*^)	0.682	0.466
185	I spend hours exercising to be in control of my weight. (^*^)	0.539	0.291
186	Eating is a sign of weakness. (^*^)	0.696	0.485
187	Controlling my eating makes me feel in control of everything. (^*^)	0.870	0.757
188	I find it easier to focus on obsessive thoughts and rituals than to think about things in my life that feel out of control. (^*^)	0.797	0.635
189	Eating only ‘healthy’ foods makes me feel cleaner and purer. (^*^)	0.582	0.338
190	Controlling my eating stops me being too needy. (^*^)	0.763	0.582

*Note*.

(*) *Item specifically designed to assess the schema mode in eating disorders context*.

(¤) *Item taken from the original SMI*.

The Self Aggrandizer mode was adapted by adding one item that specifically mentions using thinness as a way of feeling superior to others. In addition, an existing item was adapted to include thinness as a way of being “Number One,” where the original scale just referred to success, popularity, wealth and power. These adaptations were designed to highlight the specific ways in which ED behaviors may be used by some to compensate for an underlying sense of inadequacy, whereby their superiority in this specific regard is perceived as protection from future risk of humiliation or shame. Bully and Attack mode largely consisted of items from the original SMI, with three additional items generated by patients with EDs who were involved in developing the SMI-ED, linked to the need to be on guard and outsmart others to ensure that they don't take advantage. When piloting the new scale, patients indicated that they preferred the wording of “I am invincible” to “I am invulnerable,” even though the concept is basically similar. The patients identified with the concept of invincibility as a coping solution to avoid underlying vulnerability. Controlling and dominating others is also used as a way of achieving invincibility (to mask underlying vulnerability). This concept of invincibility is a thread that seems to connect all overcompensatory coping modes in eating disorders.

The Helpless Surrenderer Mode included 5 new items, and is conceptualized as a “surrender” coping mode whereby the person feels dependent on others to meet their emotional needs without taking the risk of explicitly expressing authentic vulnerability. This mode has a feel of helplessness, whereby the person wants others to understand them, whilst being resigned to the belief that others will never meet their needs (“I need people to listen to me and make me feel better”; “I want people to understand me without me having to say anything”). This coping mode appears to be driven by an acute underlying sense of feeling emotionally deprived whereby needs for emotional attunement, empathy and guidance have not been adequately met. In this mode, they often feel “stuck” and unable to find other [healthy] ways of coping, due to an overwhelming sense of impotence (“It's too hard to make changes to my behavior”). In this mode, the person may cling and act in a possessive way to cope with the pain of the unmet need, but feels unable to ask for emotional needs to be met in an open or direct way (“I am very possessive and hold on to the people that are important to me”). They express frustration that others won't provide solutions and relief from emotional suffering, whilst avoiding genuine engagement and connection (“I feel angry and desperate when people can't see I need help”) (Simpson, 2016).

Through a screening process highly similar items were identified; those items most representative of the ED population under consideration were retained and any redundant questions removed. This resulted in a 190 item SMI-ED with 16 different modes clustered thematically (Table 2): (A) five innate child modes; (B) two maladaptive [internalized/introject] modes; (C) seven maladaptive coping modes; and (D) two healthy factors. Specifically, the three child modes are: (1) Vulnerable Child—VC; (2) Angry Child– AC; (3) Enraged Child—EC; (4) Impulsive Child—IC; and (5) Undisciplined Child—UC. The two maladaptive (internalized/introject) modes are: (6) Punitive mode—PM and (7) Demanding Mode—DM. The 12 modes were comprised of both original and new statements. Two modes (Impulsive Child—IC and Enraged Child—EC) only included items retrieved from the original version of the SMI, while the Helpless Surrenderer—HS and the Eating Disorder Overcontroller—EDO modes exclusively consisted of new ED-specific statements. For each item, participants responded on a 6-point scale ranging from 1 (“never or hardly ever”) to 6 (“all of the time”) regarding how often they believe or feel the statement to be true for them. The items of the SMI-ED were presented clustered by mode rather than in random order. In fact, while it has been previously argued that randomizing items prevents response bias (Lobbestael et al., 2010), recent findings indicated that clustering items results in more accurate measurement of schema modes as it allows participants to reflect upon different aspects of themselves (Marais et al., 2017). Within each mode the items were randomized. Administration time for the SMI-ED was approximately 30 minutes.

The seven maladaptive coping modes are: Compliant Surrenderer—CS; (9) Helpless Surrenderer—DS; (10) Detached Protector—Det.P; (11) Detached Self-Soother—Det.S-S; (12) Self Aggrandizer—SA; (13) Bully and Attack—BA; (14) Eating Disorder Overcontroller—EDO. Finally, the functional healthy modes are: (15) Happy Child—HC and (16) Healthy Adult—HA.

The number of items between scales varies from 5 (Helpless Surrenderer–DS) to 20 (Vulnerable Child–VC). To compute the scale score for each mode, the scale sum score is divided by the number of items in that scale. The higher the score, the more frequent were the manifestations of the modes. No total score was computed.

### Procedure

This study was completed entirely online and was hosted by the questionnaire tool Survey Monkey. Recruitment advertisements included a link to the information page where participants provided informed consent before commencing. At the completion of the questionnaire, participants were directed to a debrief page where they were provided with contact details for support services. This study was approved by the Human Research Ethics Committees at the University of South Australia.

### Statistical analyses

Confirmatory Factor Analysis (CFA) was performed using “lavaan” package (Rosseel, 2012; Rosseel et al., 2015) for R software [R-core project (Team RC., 2014, 2015) in order to test the factorial structural model of the SMI-ED based on prior empirical and theoretical grounds. All the other statistical analysis were carried out with SPSS software (version 20.0, SPSS Inc., Bologna, Italy) (Nie et al., 1970).

### Preparation of data for statistical analysis

Items' descriptive statistics revealed a non-perfect normal distribution of some indicators. No missing data was detected. Consequently, the Robust Maximum Likelihood method (MLM) (Bentler, 1995; Muthén et al., 1997; Muthén and Muthén, 1998; Hoyle, 2012) was used to estimate the model via CFA. The MLM is a “robust” variant of Maximum likelihood (Bentler, 1995) that provides robust standard errors and a Satorra-Bentler test statistic (Satorra and Bentler, 1988, 1994; Rosseel, 2012). Factor loadings were tested for statistical significance, setting the level of *p* at 0.005. The ratio of χ^2^ to the degrees of freedom (*df*) (Jöreskog, 1969), the Comparative Fit Index (CFI) (Bentler, 1990), the Root-Mean Square Error of Approximation (RMSEA) (Steiger and Lind, 1980; Steiger, 1990) and the Standard Root Mean square Residual (SRMR) (Bentler, 1995) were also used to assess the model fit (Barrett, 2007). A non-significant χ^2^ is desirable (Bentler and Bonett, 1980) and smaller χ^2^ values indicate a better model fit. The χ^2^/*df* ratio is considered as an easily computed measure of fit (Marsh and Hocevar, 1985; Hoyle, 2012), and a χ^2^/*df* ratio value of 3 or less indicates good fit (Wheaton et al., 1977). Comparative Fit Index (CFI) designates the amount of variance and covariance accounted by the model compared with a baseline model, without sample size dependence; values higher than 0.90 are considered good/adequate (Hu and Bentler, 1999). However, Kenny and McCoach (2003) mathematically demonstrate that the number of variables being analyzed negatively affects this fit index (Russell, 2002; Kenny and McCoach, 2003; Iacobucci, 2010). The RMSEA expresses fit per degrees of freedom of the model, with values lower than 0.08 suggesting an acceptable model fit (Hu and Bentler, 1999) and values below 0.05 indicating a good fit (Browne and Cudeck, 1990). The SRMR, derives from the residual correlation matrix, and represents the average discrepancy between correlations observed in the input matrix and those predicted by the model (Bentler, 1995; Brown, 2015). A cutoff value higher than 0.8 is considered good (Hu and Bentler, 1999; Hoyle, 2012). Cronbach's alpha was computed to measure internal consistency for each SMI-ED subscale with values al least of 0.7 deemed acceptable (Cronbach, 1951). In addition, Multivariate Analysis of Covariance (MANCOVA) was conducted to assess for possible statistical differences between the disordered eating subgroups simultaneously, as measured by the (EDE-Q), on the SMI-ED subscales, while adjusting for differences on age and past and/or present consultation with a Mental Health professional as possible confounding variables.

## Results

Item analysis revealed a non-perfect normal distribution, with Kolmogorov-Smirnov and Shapiro-Wilk tests being significant (*p* < 0.001). Skewness ranged between −1.14 and 3.87 (mean_sk_ = 0.23, SD_sk_ = 0.78; mean_sk/std.err_ = 2.24; SD_sk/std.err_ = 7.61), and kurtosis ranged between −1.47 and 18.1 (mean_k_ = −0.22, SD_k_ = 2.10; mean_k/std.err_ = −1.05; SD_k/std.err_ = 10.32).

Results from the CFA suggest an acceptable sixteen-related-factors solution for the SMI-ED, despite not all the fix indexes achieving the desired value (Hu and Bentler, 1999). Indeed, the chi-square model for fit was statistically significant [$\chi_{\left(17645\right)}^{2}$ = 38210.465; *p* < 0.001] whilst the Comparative Fit Index value did not meet the threshold suggesting ideal fit [CFI > 0.90; (Hu and Bentler, 1999): CFI = 0.767]. However, the RMSEA showed a good approximation fit of the model to the data [RMSEA = 0.045 (*90% CI*: from 0.044 to 0.046), *p* (RMSEA < 0.05)= 1]. Moreover, by dividing the χ^2^ for the degrees of freedom (*df*) of the model (Jöreskog, 1969; Hu and Bentler, 1999), the proposed model is potentially acceptable (χ^2^/*df* = 2.16; <3) (Hoyle, 2012). Finally, the SRMR further suggested the goodness of model fit [SRMR = 0.077 (Hu and Bentler, 1999)].

As shown in Table 2 each item loaded significantly on the factor associated with it (*p* < 0.001), mean_loadings_ = 0.727; *SD*_loadings_ = 0.110; ranging from 0.350 (item #117) to 0.905 (item #85). In addition, correlations between the sixteen factors ranged from 0.082 to 0.838; mean_r−*factors*_ = 0.258; *SD*_*r*−factors_ = 0.404 (Table 3).

**Table 3:** Means, standard deviations and correlations between subscales of the Schema Mode Inventory for eating disorders (SMI-ED).

	**M**	**SD**	**VC**	**AC**	**EC**	**IC**	**UC**	**HC**	**PM**	**DM**	**HA**	**CS**	**Det**.	**DSS**	**SA**	**BA**	**HS**	**EDO**
VC	3.87	1.22	–															
AC	2.85	1.21	0.670	–														
EC	1.73	0.88	0.395	0.691	–													
IC	2.55	1.22	0.461	0.611	0.646	–												
UC	3.05	1.16	0.415	0.498	0.443	0.574	–											
HC	2.83	0.98	−0.745	−0.550	−0.359	−0.330	−0.377	–										
PM	3.57	1.47	0.838	0.546	0.331	0.436	0.314	−0.656	–									
DM	4.53	1.11	0.626	0.383	0.170	0.252	0.082^*^	−0.398	0.669	–								
HA	3.21	0.84	−0.614	−0.445	−0.320	−0.341	−0.445	0.766	−0.589	−0.270	–							
CS	4.07	1.08	0.656	0.369	0.153	0.246	0.252	−0.573	0.667	0.670	−0.463	–						
Det.	3.48	1.15	0.760	0.618	0.396	0.461	0.468	−0.687	0.719	0.531	−0.562	0.599	–					
Det.S-S	3.73	1.15	0.623	0.421	0.204	0.355	0.153	−0.437	0.610	0.619	−0.275	0.535	0.520	–				
SA	2.98	1.04	0.307	0.497	0.381	0.425	0.282	−0.140	0.276	0.415	−0.103^*^	0.192	0.350	0.337	–			
BA	1.91	0.83	0.221	0.503	0.466	0.457	0.385	−0.156	0.192	0.196	−0.084^*^	0.127	0.309	0.249	0.669	–		
HS	3.69	1.15	0.562	0.614	0.445	0.299	0.450	−0.453	0.472	0.408	−0.367	0.463	0.546	0.422	0.527	0.431	–	
EDO	3.84	1.31	0.639	0.444	0.253	0.373	0.155	−0.375	0.643	0.674	−0.274	0.515	0.532	0.678	0.511	0.320	0.464	–

All correlations are significant at p < 0.001, except for

* *(p < 0.05)*.

The scales had acceptable internal consistency, with coefficients ranging from 0.807 to 0.976; mean_α−*factors*_ = 0.914; *SD*_α−factors_ = 0.048. Specifically, the highest value was registered for the Punitive Mode subscale (0.976), followed by the Vulnerable Child (0.956), Impulsive Child (0.950), Angry Child (0.948), Demanding Mode (0.947), Eating Disorder Overcontroller (0.946), Happy Child (0.933), Detached Protector (0.932), Compliant Surrender (0.926), Enraged Child (0.915), Undisciplined Child and Healthy Adult (0.908), Self Aggrandizer and Bully and Attack (0.883), Helpless Surrenderer (0.808), and Detached Self-Soother (0.807).

### Concurrent validity: correlation between SMI-ED factors and eating disorder variables

Bivariate correlations calculated with direct scores indicated that all SMI-ED factors were significantly correlated (ranging from low to high levels) with EDE-Q subscales and eating disorder symptoms (Table 4). The adaptive modes (Happy Child and Healthy Adult) were negatively associated with all of the eating disorder variables.

**Table 4:** Correlations between SMI-ED' subscales, ED Symptoms (†) and EDE-Q subscales (‡).

	**^†^Times OE**	**^†^Times** **Bingeing**	**^†^Days** **Bingeing**	**^†^Vomit**	**^†^Laxatives**	**^†^Exercise**	**Restraint**	**Eating** **Concerns**	**Shape** **Concerns**	**Weight** **Concerns**	**Global**
VC	0.130	0.213	0.197	0.220	0.231	0.283	0.579	0.741	0.686	0.694	0.758
AC	0.069^§^	0.140	0.141	0.157	0.243	0.191	0.334	0.452	0.369	0.436	0.454
EC	0.114^*^	0.181	0.180	0.140	0.236	0.167	0.201	0.256	0.205	0.234	0.253
IC	0.223	0.272	0.254	0.265	0.267	0.174	0.311	0.369	0.321	0.337	0.377
UC	0.232	0.260	0.255	0.206	0.202	−0.030^§^	0.133	0.287	0.250	0.265	0.260
HC	−0.031^§^	−0.093^*^	−0.084^*^	−0.163	−0.204	−0.196	−0.378	−0.461	−0.424	−0.442	-479
PM	0.094^*^	0.165	0.105^*^	0.244	0.237	0.308	0.567	0.675	0.601	0.617	0.692
DM	0.063^§^	0.125	0.061^§^	0.154	0.161	0.307	0.533	0.549	0.557	0.540	0.613
HA	−0.060^§^	−0.089^*^	−0.087^*^	−0.174	−0.191	−0.079^§^	−0.278	−0.405	−0.395	−0.413	−0.416
CS	0.125	0.170	0.132	0.181	0.168	0.267	0.447	0.519	0.508	0.512	0.557
Det.	0.069^§^	0.135	0.146	0.205	0.229	0.249	0.475	0.551	0.481	485	0.561
Det.S-S	0.121	0.182	0.130	0.176	0.174	0.362	0.469	0.509	0.471	0.473	0.541
SA	−0.008^§^	0.011^§^	0.029^§^	0.084^*^	0.163	0.180	0.244	0.235	0.216	0.225	0.260
BA	0.028^§^	0.060^§^	0.106^*^	0.036^§^	0.155	0.107^*^	0.156	0.150	0.107^*^	0.135	0.156
HS	0.071^§^	0.119^*^	0.126	0.162	0.152	0.160	0.274	0.372	0.367	0.391	0.392
EDO	0.038^§^	0.122	0.055^§^	0.196	0.214	0.419	0.628	0.644	0.575	0.576	0.685

Note. All correlations are significant at p < 0.001, except for

* (p < 0.020) and

§ (p > 0.05; ns). Times OE: Over the past 28 days, how many TIMES have you eaten what other people would regard as an unusually large amount of food (given the circumstances)?—Times bingeing: On how many of these TIMES did you have a sense of having lost control over your eating (at the time that you were eating)?—Days bingeing: Over the past 28 days, on how many DAYS have such episodes of overeating occurred (i.e., you have eaten an unusually large amount of food and have had a sense of loss of control at the time)?—Vomit: Over the past 28 days, how many TIMES have you made yourself sick (vomit) as a means of controlling your shape or weight?—Laxatives: Over the past 28 days, how many TIMES have you taken laxatives as a means of controlling your shape or weight?—Exercise: Over the past 28 days, how many TIMES have you exercised in a “driven” or “compulsive” way as a means of controlling your weight, shape or amount of fat, or to burn off calories?

### Mode scores across disordered eating subscales

While controlling for age and past and/or present consultation with a Mental Health professional as possible confounding variables, Multivariate Analysis of Covariance (MANCOVA) revealed a significant difference, between disordered eating behaviors (EDE-Q subscales) on the majority of the SMI-ED subscales: Wilks's Λ = 0.693, *F* = 4.473, *p* < 0.001, η^2^ = 115. No differences emerged between the EDE-Q subscales for the Healthy Adult, Self-Aggrandizer, Bully and Attack, and Helpless Surrenderer modes as measured by the SMI-ED. Also, in order to test differences between groups within SMI-ED subscales, ANCOVAs with focused contrasts were conducted for each dependent variable. Results are shown in Table 5. Mean scores are shown for each EDE-Q subscale in relation to each of the schema modes. In particular, in relation to the “new” modes (Helpless Surrenderer and Eating Disorder Overcontroller), means were above the 75% percentile rank for young adult women (Mond et al., 2006) except for the Shape Concern Subscale (particularly for the Overcontroller), demonstrating the adequacy of these modes in reflecting eating disorder behaviors. In particular, the means of the new subscales were higher for Restraint, reflecting the high degree of restrictive behavior and preoccupation with food in this sample. The “Restraint” and “Eating Concern” subscales of the EDE-Q discriminated the majority of modes most representative of eating disorder populations (i.e., Impulsive Child, Punitive Parent, Demanding Parent, Detached Self-Soother, Compliant Surrenderer and Eating Disorder Overcontroller). Only the Vulnerable Child Mode did not show significant contrasts between EDE-Q subscales. In the case of Demanding and Punitive Modes, the “Restraint” subscale also significantly discriminated at a higher level than the “Eating Concern” subscale. In particular, focused contrasts indicated that “Restraint” and “Eating Concern” subscales significantly discriminated the following modes at a higher level than the “Shape-” and “Weight concern” subscales: Eating Disorder Overcontroller, Compliant Surrenderer, Detached Self-Soother, Demanding Parent and Punitive Parent Modes. Both Restraint and Eating Concern subscales significantly discriminated the Impulsive Child Mode over and above the Weight Concern subscale.

**Table 5:** Mean (SD) for the ED subscale, results of Multivariate Analysis of Covariance.

	**Restraint** **(*n* = 130)**	**Eating Concern** **(*n* = 298)**	**Shape Concern** **(*n* = 77)**	**Weight Concern** **(*n* = 68)**			
	**Mean (SD)**	**Mean(SD)**	**Mean(SD)**	**Mean(SD)**	***F* (3,567)**	**η^2^**	**Focused contrast^a^**
VC	4.29 (1.09)	4.07 (1.23)	3.26 (1.31)	2.92 (1.01)	22.965	0.109	1>3; 1>4; 2>3; 2>4
AC	2.95 (1.36)	2.95 (1.18)	2.63 (1.22)	2.43 (0.91)	2.775^**^	0.014	1>4^**^; 2>4^*^
EC	1.76 (0.88)	1.79 (0.93)	1.69 (0.846)	1.48 (0.59)	2.191	0.011	1>4^**^; 2>4^**^
IC	2.53 (1.26)	2.74 (1.28)	2.31 (1.04)	2.04 (0.83)	5.711^*^	0.029	1>4^**^; 2>4^*^
UC	2.89 (1.19)	3.17 (1.13)	3.25 (1.25)	2.61 (1.01)	6.628	0.034	1 <2^**^; 1 <3^**^; 2>4^*^; 3>4^*^
HC	2.57 (0.93)	2.77 (0.94)	3.11 (1.03)	3.26 (0.95)	5.264^*^	0.029	1 <3^*^; 1 <4^**^; 2 <4^*^
PM	4.28 (1.41)	3.71 (1.41)	2.71 (1.27)	2.54 (1.25)	24.500	0.115	1>2^*^; 1>3^*^; 1>4^*^; 2>3^*^; 2>4^*^
DM	4.97 (0.92)	4.56 (1.01)	3.95 (1.25)	4.03 (1.21)	13.174	0.065	1>2^*^; 1>3^*^; 1>4^*^; 2>3^*^; 2>4^*^
HA	3.03 (0.82)	3.17 (0.83)	3.35 (0.85)	3.49 (0.81)	2.526^§^	0.013	1 <4^*^; 2 <4^**^
CS	4.41 (1.11)	4.15 (1.01)	3.74 (1.06)	3.45 (1.01)	10.137	0.051	1>3^*^; 1>4^*^; 2>3^**^; 2>4^*^
Det.	3.84 (1.09)	3.61 (1.09)	2.93 (1.21)	2.86 (0.96)	13.302	0.066	1>3^*^; 1>4^*^; 2>3^*^; 2>4^*^
Det.S-S	3.99 (1.18)	3.93 (1.03)	1.13 (1.12)	3.06 (1.14)	15.411	0.076	1>3^*^; 1>4^**^; 2>3^*^; 2>4^*^
SA	3.07 (1.16)	3.03 (1.02)	2.69 (0.94)	2.94 (0.91)	0.865^§^	0.005	–
BA	1.84 (0.79)	1.98 (0.86)	1.89 (0.83)	1.79 (0.75)	1.382^§^	0.007	–
HS	3.73 (1.17)	3.81 (1.14)	3.45 (1.13)	3.42 (1.12)	1.718^§^	0.009	2>4^**^
EDO	4.31 (1.29)	4.11 (1.45)	2.72 (1.11)	3.11 (1.15)	32.754	0.148	1>3^*^; 1>4^*^; 2>3^*^; 2>4^*^; 3>4^**^

All contrasts are significant at p <0.001, except for

* *(p <0.010)*,

** (p <0.050) and

§ *(p > 0.05; ns)*.

a *Focused contrast with covariates (ANCOVAs) was performed in order to test potential differences between EDE-Q subscales (1. Restraint; 2. Eating concern; 3. Shape Concern; 4. Weight concern) within the SMI-ED dimensions. “Age” and “past and/or present consultation with a Mental Health professional” were used as covariates*.

## Discussion

This study tested the psychometric properties of the SMI-ED, an adapted version of the original SMI, and investigated its psychometric properties within a population characterized by disordered eating patterns. Findings indicated an adequate fit for a 16-factor model, with moderate intercorrelations between subscales, and high internal consistency within subscales.

Although the chi-square model for fit and the CFI values resulted in a less than ideal fit, it must be highlighted that fit indices are affected by the sample size [i.e., chi-square (Jöreskog, 1969; Tuker and Lewis, 1973; Bentler and Bonett, 1980; James and Mulaik, 1982; Kline, 2015)] and the number of considered indicators respectively [i.e., CFI (Hu and Bentler, 1998; Russell, 2002; Kenny and McCoach, 2003; Fan and Sivo, 2005; Iacobucci, 2010; Kline, 2015)]. Indeed, as suggested by Kenny and McCoach (2003), testing well-fitting models with too many indicators, may cause malfunctioning in the CFI index, but it should not be a cause for concern if the SRMR and RMSEA reveal a good approximation fit of the model to the data (Hu and Bentler, 1998, 1999; Russell, 2002; Kenny and McCoach, 2003; Fan and Sivo, 2005; Iacobucci, 2010; Kline, 2015).

The final SMI-ED comprised 190 items that were found to best represent and distinguish 16 schema modes within this sample (the Helpless Surrenderer-HS and the ED-Overcontroller—EDO modes were specifically added for adapting the tool to the EDs sample). The final 16 factors indicated 5 maladaptive child modes; 2 maladaptive internalized/introject modes; 7 maladaptive coping modes; and 2 healthy factors, and included 73 new items. This supports our suggestion that the original SMI may not adequately capture the mode presentations for EDs, and that they may be better represented by the inclusion of items more directly related to the experiences reported by this specific population. The potential utility of measuring schema modes using separate subscales is therefore confirmed. The addition of two new coping modes, as well as additional items added to existing mode scales within the SMI-ED are likely to be of clinical utility in facilitating identification of ED-specific behaviors that characterize specific modes. Although mode scale scores may remain relatively unaffected by the new items, the qualitative examination of specific high-scoring items is likely to be of clinical value.

In support of Lobbestael et al.'s (2010) original findings (Lobbestael et al., 2010), Vulnerable, Angry, Enraged, Undisciplined, Impulsive, and Contented Child modes and the two “parent” (or introject) modes retained some items from the original SMI, with the addition of some new items. The ED-Overcontroller Mode (EDO) was found to be differentiated in the SMI-ED, supporting the notion that what was previously identified as a clinically important concept forms a measurable and coherent mode (Simpson, 2012, 2016) and upholding previous findings (Brown et al., 2016). All of these were new items. This mode is overcontrolling of the self and/or others and uses over-control of the body and perfectionism, to overcompensate for an underlying sense of powerlessness, shame or guilt. This overcompensatory coping mode can lead to feelings of mastery, powerfulness and pseudo-control (Arntz, 2010; Simpson, 2012, 2016). Results indicated that the one new item and one adapted item in Self Aggrandizer mode clustered with the 9 items that were retained from this mode within the original SMI. This supports our clinical observations that this mode may take on a slightly different “flavour” to the Self Aggrandizer in the original SMI, as the emphasis is on extreme high standards and a sense of pride or even superiority, often with an excessive emphasis on shape, weight and thinness which is perceived to provide protection from further shame or criticism (Simpson, 2016). This is consistent with findings for those with Obsessive-Compulsive Personality Disorder, who in this mode experience themselves as superior to others who are perceived as less principled and scrupulous (Beck et al., 2004; Weertman et al., 2008; Bamelis et al., 2015). Two of the three new items proposed for the Bully and Attack mode (“I feel invincible” and “If I'm not constantly on guard people will take advantage of me”) did not have a high loading on this scale, and will therefore be removed from the shorter scale in the second study. This may be because they are both likely to function as passive mechanisms for defending themselves against possible attack from others, rather than overtly bullying others.

The Helpless (or “Aggrieved”) Surrenderer Mode included 5 new items, and all but one had high loadings on this factor. The lower loading on the item “I am very possessive and hold on to the people that are important to me” may be indicative of the pattern of passivity and resignation connected with this mode, whereby the person feels helpless to find direct ways of asking for support and connection from others. This mode is characterized by a pattern whereby others are expected to intuitively know what they feel and need, with a tendency to feel frustrated when this does not materialize (Simpson, 2016). This mode has some overlap with the Complaining Protector mode (Bernstein and van den Broek, 2009) and also perhaps the Self-Pity/Victim Mode (Edwards, 2012, 2015) in the sense that all of these modes are represented by a passive-aggressive style of communication and a tendency to externalize responsibility for getting one's needs met. In this mode, self-deprivation, and physical frailty become an indirect means of communicating a need for emotional support (e.g., “if I am physically unwell, they will see how much I need them”). The remaining coping modes (Compliant Surrenderer, Detached Protector, Detached Self-Soother) and Healthy Adult mode were parallel to those described in the original validation study (Lobbestael et al., 2010) with the addition of some new items.

Correlations between modes were generally high. As expected, correlations between Punitive and Demanding Parent modes and Vulnerable and Angry Child modes were high, reflecting the toxic effect of internalized critical messages in generating emotional distress. Detached Protector and ED-Overcontroller were also surprisingly highly correlated with Vulnerable Child mode, which may be an indication of mode-flipping, whereby one closely follows the other. Indeed, coping modes are generally utilized to keep Vulnerable child mode (and attendant distress) outside conscious awareness. There was a significant difference between disordered eating behaviors (EDE-Q subscales) on the majority of the SMI-ED subscales, with most indicating higher levels of restraint and eating concern compared with shape and weight concern (Table 5). Both “Restraint” and “Eating Concern” subscales of the EDE-Q discriminated the modes most representative of eating disorder populations. In the case of the “Parent” (Introject) modes “Restraint” discriminated these more significantly than “Eating Concern” subscale. The higher mean scores on the “Restraint” and “Eating Concern” subscales compared with the “Shape-”and “Weight-Concerns” subscales may be due to the high number of participants with high levels of restrictive behaviors in this sample (as indicated by endorsement of: restraint over eating, avoidance of eating, food avoidance, dietary rules and desire for an empty stomach). Indeed, “Restraint” remained significantly higher than “Eating Concern” in spite of the fact that it comprised less than half of the number of participants (compared with “Eating Concern”), further corroborating this result. “Restraint” represents an attitude and behaviors that are highly relevant across all eating disorders. It is the main symptom that perpetuates AN, precedes binges in bulimic disorders, and also functions as a compensatory behavior in BN. In addition, the mean BMI in the “Restraint” category was low (18.18), indicating the majority of participants were characterized by restrictive eating patterns.

As expected, in this clinical sample the majority of eating disorder symptoms were positively correlated with Internalized/Introject and Coping modes and negatively correlated with the Healthy modes. Three of the coping modes, Bully-Attack, Self-Aggrandizer and Helpless Surrenderer only showed low correlations with eating disorder symptoms. It may be that these modes are more relevant to maintenance cycles or to other co-morbid symptoms, rather than directly influencing specific ED symptoms. Alternatively, it seems plausible that as the majority of these modes are described for a wide range of disorders, they may have a stronger association with general psychopathology than specific symptoms associated with eating disorders. Future studies may explore the role of these modes further within the ED population, and investigate whether they are more relevant to specific ED diagnostic groups. However, there were moderate to high correlations between the majority of coping modes and restraint and eating/weight/shape concerns. This is consistent with previous studies which have highlighted the link between avoidant coping, numbness, feelings of “lightness” and even euphoria, and restrictive eating/starvation syndrome (Spranger et al., 2001; Wildes et al., 2010; Kaye et al., 2013; Brown et al., 2016). The fact that these coping modes emerged as significant factors is consistent with findings from previous studies (Talbot et al., 2015; Brown et al., 2016). Low correlations were identified between other specific ED symptoms (e.g., bingeing, vomiting, exercise) and schema modes, however correlations between global eating disorder ratings and schema modes were mostly moderate to high.

### Practical implications

The adapted SMI-ED was developed to increase the relevance of the measure for an ED population. At this preliminary stage of development, this measure is likely to be of potential clinical utility both quantitatively and qualitatively. In fact, the two new schema coping modes—the Eating Disorder Overcontroller and Helpless Surrenderer modes—provide additional information regarding coping styles that are specific of the ED population, still not available from the original SMI. The adaptation of the SMI for EDs offers an opportunity for clinicians to identify and explore a wider range of mechanisms through which modes are expressed in this population. In turn, this will facilitate the development of individually tailored case conceptualizations and treatment. It is hoped that the scale will enable a greater shared understanding of ED difficulties within treatment settings, and will give those with EDs greater therapeutic opportunities for making important links between eating disorder symptoms/behaviors, and schema modes.

### Limitations and recommendations for future research

Given the large number of items in the SMI-ED, it is questionable whether the sample was large enough to allow for definitive findings. This survey was specifically developed to identify participants with disordered eating, including those with subthreshold eating disorders, and therefore is not directly generalizable to a clinical population. In addition, as this was an online survey that asked participants to take part if they identified themselves as having disordered eating behaviors, there is likely to have been some self-selection bias, with an under-representation of those who were “pre-contemplative” (i.e., characterized by a reduced capacity to recognize and/or acknowledge their eating disordered behaviors). It is also possible that those with more severe eating disorders who may be more isolated, and/or avoidant of eating disorder clinical and support services may have been under-represented. Furthermore, as the sample was purely recruited via online survey in a naturalistic setting, it was not possible to ensure gender homogeneity among respondents. Still, although only a small proportion of the sample was male, this is representative of the gender ratio found in clinical settings (Striegel-Moore et al., 2009). In addition, the use of self-report measures did not allow diagnosis, nor a comprehensive assessment of eating disordered symptoms. A relatively low proportion of participants endorsed binge eating behaviors compared with other dysfunctional eating patterns. Future studies should ideally include a larger percentage of males in the sample. In addition, diagnostic measures should be utilized to ensure that all ED subgroups are adequately represented within the sample, so as to ascertain whether particular profiles of schema modes exist within specific diagnostic groups. However, given that it is becoming increasingly recognized that a transdiagnostic approach is more appropriate for capturing the complexities of individual presentations, it may be that the complex interplay between personality disorders and eating disorder diagnoses will make this a difficult task. Indeed, previous studies have identified that schema modes may be more strongly explained by strength of personality disorder than non-characterological (Axis I) disorders (Lobbestael et al., 2010). This highlights the potential importance for future researchers and clinicians to measure personality disorder alongside eating disorder symptomatology.

This study represents a preliminary step in understanding the schema modes represented within the ED population. An independent replication and assessment of test-retest reliability and construct validity across the range of eating disorder diagnostic groups is required. There is a need for further differentiation of modes within specific ED diagnostic groupings, and the degree to which this is statistically viable. Finally, future studies are needed to explore the psychometric properties and factorial structure of the SMI-ED in both clinical and non-clinical populations, across other countries and languages, as well as to identify whether a shortened version of the scale is feasible.

## Conclusion

This study represents the first step in creating a psychometrically sound instrument for the assessment of schema modes in EDs, and provides greater insight for the conceptualization and treatment of this population. This measure was comprised of a hybrid of 117 items from the original SMI with 73 new eating-disorder-specific items relating to schema modes. Confirmatory Factor Analysis confirmed the sixteen-related-factors solution for the 190-item SMI-ED, thus providing preliminary evidence for the adequate reliability of the new measure. The adaptation of the SMI for those with disordered eating represents the first step in developing a tailor-made measure of schema modes for the ED population. The adapted SMI-ED provides a tool for identifying and exploring a wide range of mechanisms through which modes are manifested in this population. Further studies are warranted to further refine and shorten the SMI-ED, in order to improve its utility as a research and clinical tool with this population.

## Author contributions

SS conceived the study and wrote the manuscript with support from GP and AR. GP also contributed to data interpretation, and AR performed the analysis. GM helped supervise the project. TS contributed to data collection and preparation. CM and JN revised the advanced versions of the manuscript. GC approved the final version of the manuscript.

### Conflict of interest statement

The authors declare that the research was conducted in the absence of any commercial or financial relationships that could be construed as a potential conflict of interest.

## Abbreviations

EDs: Eating Disorders
CBT: Cognitive Behavioral Therapy
BN: Bulimia Nervosa
BED: Binge Eating Disorder
AN: Anorexia Nervosa
CBT-E: Enhanced CBT
ST: Schema Therapy
EMS: Early Maladaptive Schemas
SMI-ED: Schema Mode Inventory for Eating Disorders
VC: Vulnerable Child
AC: Angry Child
EC: Enraged Child
IC: Impulsive Child
UC: Undisciplined Child
PM: Punitive Mode
DM: Demanding Mode
CS: Compliant Surrender
Det.P: Detached Protector
Det.S-S: Detached Self-Soother
S-A: Self Aggrandizer
BA: Bully and Attack
HS: Helpless Surrenderer
EDO: Eating Disorder Overcontroller
HC: Happy Child
CFA: Confirmatory Factor Analysis
SMI: Schema Mode Inventory
SMI-ED: Schema Mode Inventory for Eating Disorders
CFI: Comparative Fit Index
RMSEA: Root-Mean Square Error of Approximation
SRMR: Standard Root Mean square Residual
MANCOVA: Multivariate analysis of covariance.
